# H_3_PMo_12_O_40_ Immobilized on Amine Functionalized SBA-15 as a Catalyst for Aldose Epimerization

**DOI:** 10.3390/ma13030507

**Published:** 2020-01-21

**Authors:** Hui Wang, Meiyin Wang, Jining Shang, Yuanhang Ren, Bin Yue, Heyong He

**Affiliations:** Department of Chemistry and Shanghai key Laboratory of Molecular Catalysis and Innovative Materials, Collaborative Innovation Center of Chemistry for Energy Materials, Fudan University, Shanghai 200438, China; 17210220053@fudan.edu.cn (H.W.); 14110220018@fudan.edu.cn (M.W.); 18110220054@fudan.edu.cn (J.S.); YuanhangRen@fudan.edu.cn (Y.R.)

**Keywords:** aldose epimerization, phosphomolybdic acid, amino-silanization, SBA-15

## Abstract

In this work various amount of phosphomolybdic acid (PMo) were immobilized on amine functionalized SBA-15 and used as heterogeneous catalysts in the epimerization of glucose in aqueous solution. 13.3PMo/NH_2_-SBA-15 exhibited the best catalytic performance with a glucose conversion of 34.8% and mannose selectivity of 85.6% within two hours at 120 °C. The activation energy of 80.1 ± 0.1 kJ·mol^−1^ was lower than that of 96 kJ·mol^−1^ over the homogeneous H_3_PMo_12_O_40_ catalyst. The catalytic activities of 13.3PMo/NH_2_-SBA-15 for the transformation of some other aldoses including mannose, arabinose and xylose were also investigated.

## 1. Introduction

**Scheme 1:**
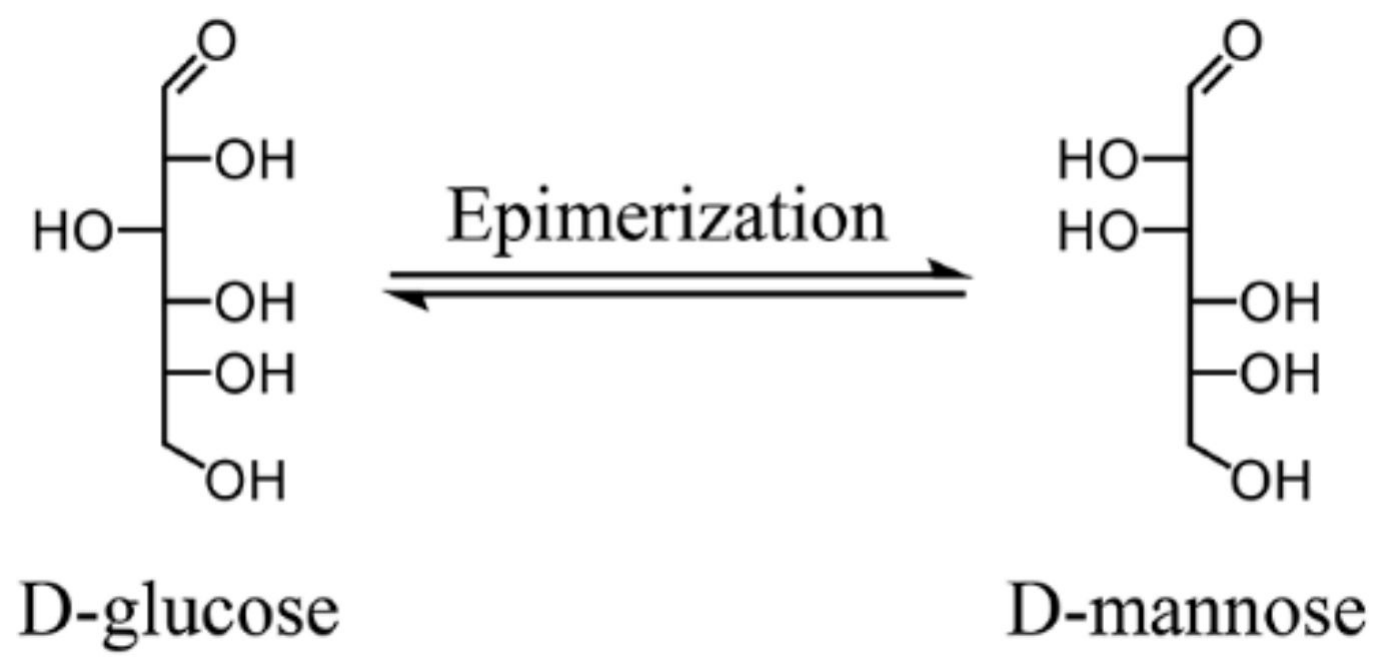
The epimerization of glucose to mannose.

As a renewable carbon resource, biomass is considered to be an ideal substitute for traditional fossil resources [1,2]. Carbohydrates are easily available in the utilization of plant biomass and glucose obviously prevails as the main building block [3,4]. As the most abundant C6 monosaccharide in nature, glucose can be easily extracted in large volumes from cellulose and hemicellulose, so many efforts have been put into valorizing glucose [5]. Among the various chemical transformations of glucose, epimerization is a carbon-efficient pathway for the production of chiral counterparts (Scheme 1) [6,7,8].

Epimerization is widely used to produce some rare sugars with valuable properties since these sugars could not be largely obtained from biopolymers. For example, sugars like D-lyxose and L-ribose, which are produced based on their abundant C-2 epimers, can be employed to synthesize antitumor agents and hepatitis B virus resistance drugs, respectively [9,10]. Currently, the large-scale production of these rare monosaccharides mainly relies on enzyme catalytic process, which suffers from the strict operating conditions such as specific temperature and pH value [11]. In addition, epimerases are only active on the monosaccharides pretreated by phosphate or nucleotide and difficult to be separated from the reaction system, so developing inorganic catalysts is becoming desirable to overcome some of these difficulties [11,12].

Lobry de Bruyn−Alberda van Ekenstein (LdB−AvE) reaction is a classic transformation for aldose epimerization with soluble alkalis as catalysts, such as NaOH and Ca(OH)_2_ [13,14]. Aldose converts into an enediol anion in a basic media and the C-2 epimer forms through the rotation of the C2-C3 bond of the intermediate [15]. Since the reorganization of the enol intermediate into isomeric ketose is faster than transformation into epimeric aldose and numerous side reactions exist in basic system, the selectivity of the epimeric aldose is extremely low. For a heterogeneous Lewis acid catalyst, Sn-β zeolite was found to simultaneously catalyze the isomerization and the epimerization of aldose in aqueous solution, but the former was always dominant [16]. Gunther et al. [17] showed that the epimeric aldose was the main product when sodium tetraborate was introduced into the catalytic system of Sn-β. Later on, Bermejo-Deval et al. [18] reported that Na^+^ exchanged Sn-β could also catalyze the epimerization of glucose in aqueous solvent. However, Na^+^ cations in the active sites could not be well preserved under the reaction condition. With increasing reaction time, the selectivity of epimerization to mannose gradually shifted to isomerization to fructose. In a word, no matter the reaction occurs in a base-catalyzed or a Lewis acid-catalyzed system, there is a competition between the isomerization and epimerization of aldose. Therefore, it is a big challenge to obtain the C-2 epimer with high selectivity. In 1970s, Bilik et al. [19,20,21] found that the epimerization reaction of aldose reached the thermodynamic equilibrium in 2–6 h at 70–90 °C with molybdate as a catalyst in acid solution, and no isomeric ketose formed during the reaction. The intramolecular 1,2 carbon shift mechanism or called as “Bilik mechanism” was then proposed, in which the C2–C3 bond of the aldose cleaves while the C1–C3 bond forms [22]. However, the epimerization reaction only proceeds rapidly when the pH value of the system is in the region of 2.5–3.5. Hence additional inorganic acid is always needed [23]. Chethana et al. [24] calculated the activation barrier and proposed that the molybdenum species are mainly in the form of dimers in the pH range of 2.5–3.5. The di-molybdate-glucose complex of great flexibility could facilitate the rearrangement of carbon skeleton. Inspired by the pioneer work of Bilik et al. [19,20,21], considerable efforts have been made to develop effective Mo-catalysts for aldose epimerization [25,26,27,28]. In 2014, Ju et al. [29] reported that H_3_PMo_12_O_40_ exhibited high activity in the epimerization of glucose, and the Bilik reaction mechanism was confirmed by ^13^C NMR spectroscopy. Polyoxometalates (POMs) can provide the active sites without utilizing additional inorganic acid and exhibit great potential in the aldose epimerization reaction. However, it is not an easy task to separate homogeneous H_3_PMo_12_O_40_ from the aqueous solution for recycling. In this case, Ju et al. [29] also tried to exchange the protons with Ag^+^ to solidify H_3_PMo_12_O_40_ catalyst. Lari et al. [30] reported that Ag_3_PMo_12_O_40_ is not a stable heterogeneous catalyst in hot aqueous solution. Even under a quite mild condition (60 °C, 0.5 h), the leaching of Mo species in Ag_3_PMo_12_O_40_ reached up to 21%. As for heterogenization of POMs, dispersing POMs on an amine functionalized support with high specific surface area is also a typical strategy [31]. On account of the acid-base interaction between –NH_2_ and heteropoly acids, it is anticipated that the leaching of active Mo species could be effectively restrained in glucose epimerization reaction.

In this work, a series of phosphomolybdic acid (PMo) immobilized on amine functionalized SBA-15 catalysts (xPMo/NH_2_-SBA-15) with different PMo contents were prepared. The catalytic performance of xPMo/NH_2_-SBA-15 was tested in the glucose epimerization in the aqueous solution. The leaching of Mo species during the transformation of glucose and the reusability of the optimal catalyst were investigated. The transformation of other aldoses such as mannose, arabinose, and xylose were also studied over the catalyst.

## 2. Materials and Methods

### 2.1. Materials

Poly(ethylene glycol)-block poly(propylene glycol)-block-poly(ethylene glycol) (P123, molecular weight ~ 5800) and D-(+)-glucose (ACS reagent) were purchased from Sigma-Aldrich, Co. (St. Louis, MO, USA). Tetraethyl orthosilicate (TEOS, SiO_2_ ≥ 28%) was purchased from Shanghai Lingfeng Chemical Reagent Co., Ltd. (Shanghai, China). Ethanol (anhydrous, ≥99.7%) was purchased from Shanghai Titan Scientific Co., Ltd. (Shanghai, China). Hydrochloric acid (36.0%~38.0%), sulfuric acid (95.0%~98.0%) and toluene (≥99.5%) were purchased from Sinopharm Chemical Reagent Co., Ltd. (Shanghai, China). (3-Aminopropyl)triethoxysilane (APTES, 99%), phosphomolybdic acid hydrate (H_3_PMo_12_O_40_·nH_2_O, AR), D-fructose (99%), D-(+)-mannose (99%), L-(+)-arabinose (≥99%), L-(+)-ribose (98%) and D-(+)-xylose (≥99%) were purchased from Shanghai Aladdin Biochemical Technology Co., Ltd. (Shanghai, China). D-(-)-lyxose (99%) was purchased from Shanghai Macklin Biochemical Technology Co., Ltd. (Shanghai, China). All the chemicals were used without further purification.

### 2.2. Preparation of Catalysts

Preparation of mesoporous SBA-15 was carried out according to the literature method [32]. Typically, P123 (4.0 g), deionized water (30 g) and 2.9 mol·L^−1^ HCl (120 g) were added into a round bottom flask, and the mixture was kept stirring for 4 h at 35 °C. TEOS (8.5 g) was added into the system, then the mixture was kept stirring for 20 h. The resulting mixture was transferred into a Teflon-lined autoclave and was then aged at 100 °C for 24 h. After that, the white product was filtered, washed three times with deionized water and dried at 60 °C overnight. Mesoporous SBA-15 was obtained after the dried sample was calcined at 550 °C for 5 h in a muffle furnace with 1 °C·min^−1^ of the heating rate.

The amine functionalized SBA-15 was prepared via a post-grafting method following the reported procedure [33]. In a typical process, SBA-15 (3.0 g) was dried overnight at 110 °C under vacuum and quickly dispersed in toluene (75 mL). The mixture was refluxed at 120 °C for 4 h to remove residual water molecules in the system. APTES (1.5 g) was dissolved in toluene (15 mL) and the solution was added dropwise to the suspension with stirring for another 4 h at 120 °C. After cooling to room temperature, the product was filtered, washed with warm ethanol three times and then dried at 60 °C. The resulting material was denoted as NH_2_-SBA-15.

The immobilization of PMo on the surface of NH_2_-SBA-15 was performed as follows. Different amount of PMo (10, 20, 30, 40, and 50 mg) were dissolved in deionized water (30 mL) to obtain a clear solution and then NH_2_-SBA-15 (0.3 g) was dispersed in the solution. The mixture was stirred at room temperature for 24 h. After centrifugation, washing three times with water and drying at 60 °C overnight, xPMo/NH_2_-SBA-15 catalyst was obtained, where x represents the weight percentage of PMo (3.3, 6.7, 10, 13.3, and 16.7 wt%) anchored on NH_2_-SBA-15 in the experimental preparation process.

### 2.3. Catalyst Characterization

Fourier transform infrared (FT-IR) spectra were measured on a ThermoFisher Nicolet iS10 infrared instrument (Waltham, MA, USA) using KBr discs. X-ray powder diffraction (XRD) patterns were recorded on a Bruker D8 Advance X-ray diffractometer (Karlsruhe, Germany) using Cu-Kα radiation with a voltage of 40 kV and a current of 40 mA. The nitrogen content of catalysts was determined with a Vario EL elemental analyzer (Analysemsysteme GmbH, Langenselbold, Germany) and the molybdenum content was measured on a Perkin Elmer 8000 inductively coupled plasma atomic emission spectrometer (ICP-AES, Waltham, MA, USA). N_2_ adsorption-desorption measurements were performed at 77 K on a Micromeritic Tristar II 3020 apparatus (Norcross, GA, USA). SBA-15 was outgassed at 200 °C for 3 h and the other samples were outgassed at 100 °C for 3 h before the measurements. The specific surface area was calculated with the BET equation. The pore size and pore volume were calculated from the desorption branch of the isotherm based on the BJH model. ^31^P magic-angle spinning nuclear magnetic resonance (MAS NMR) studies were carried out on a Bruker 400 WB AVANCE III solid-state nuclear magnetic resonance instrument (Karlsruhe, Germany), and the chemical shift was determined using 85 wt% H_3_PO_4_ as a standard.

### 2.4. Catalytic Tests

The catalytic reaction of glucose epimerization was conducted as follows: catalyst (40 mg) and 5 mL of 5 wt% aqueous solution of aldose (glucose, mannose, arabinose or xylose) were added into a 50 mL autoclave and the reactor was placed in a 120 °C oil bath with magnetic stirring. After reaction with a desired time, the reactor was cooled to room temperature with flowing cold water and the solid catalyst was separated by a 0.22 μm filter. Then the reaction solution was diluted 10 times with deionized water and analyzed on an Agilent 1100 high-performance liquid chromatography (HPLC, Santa Clara, CA, USA) with a refractive index detector. A Biorad©Aminex HPX-87 sugar column was employed at 35 °C and 5 mmol·L^−1^ H_2_SO_4_ solution was used as the mobile phase at a flow rate of 0.6 mL·min^−1^. The conversion of aldose, the yield and selectivity of the corresponding epimer were calculated as follows:

$$\mathrm{Aldose}\;\mathrm{conversion}\;=\;\frac{\mathrm{moles}\;\mathrm{of}\;\mathrm{converted}\;\mathrm{aldose}}{\mathrm{moles}\;\mathrm{of}\;\mathrm{initial}\;\mathrm{aldose}}\;\mathrm{Epimer}\;\mathrm{yield}\;=\;\frac{\mathrm{moles}\;\mathrm{of}\;\mathrm{formed}\;\mathrm{epimer}}{\mathrm{moles}\;\mathrm{of}\;\mathrm{initial}\;\mathrm{aldose}}\;\mathrm{Epimer}\;\mathrm{selectivity}\;=\;\frac{\mathrm{moles}\;\mathrm{of}\;\mathrm{formed}\;\mathrm{epimer}}{\mathrm{moles}\;\mathrm{of}\;\mathrm{converted}\;\mathrm{aldose}}$$

## 3. Results and Discussion

### 3.1. Catalyst Characterization

#### 3.1.1. FT-IR Spectroscopy

**Figure 1:**
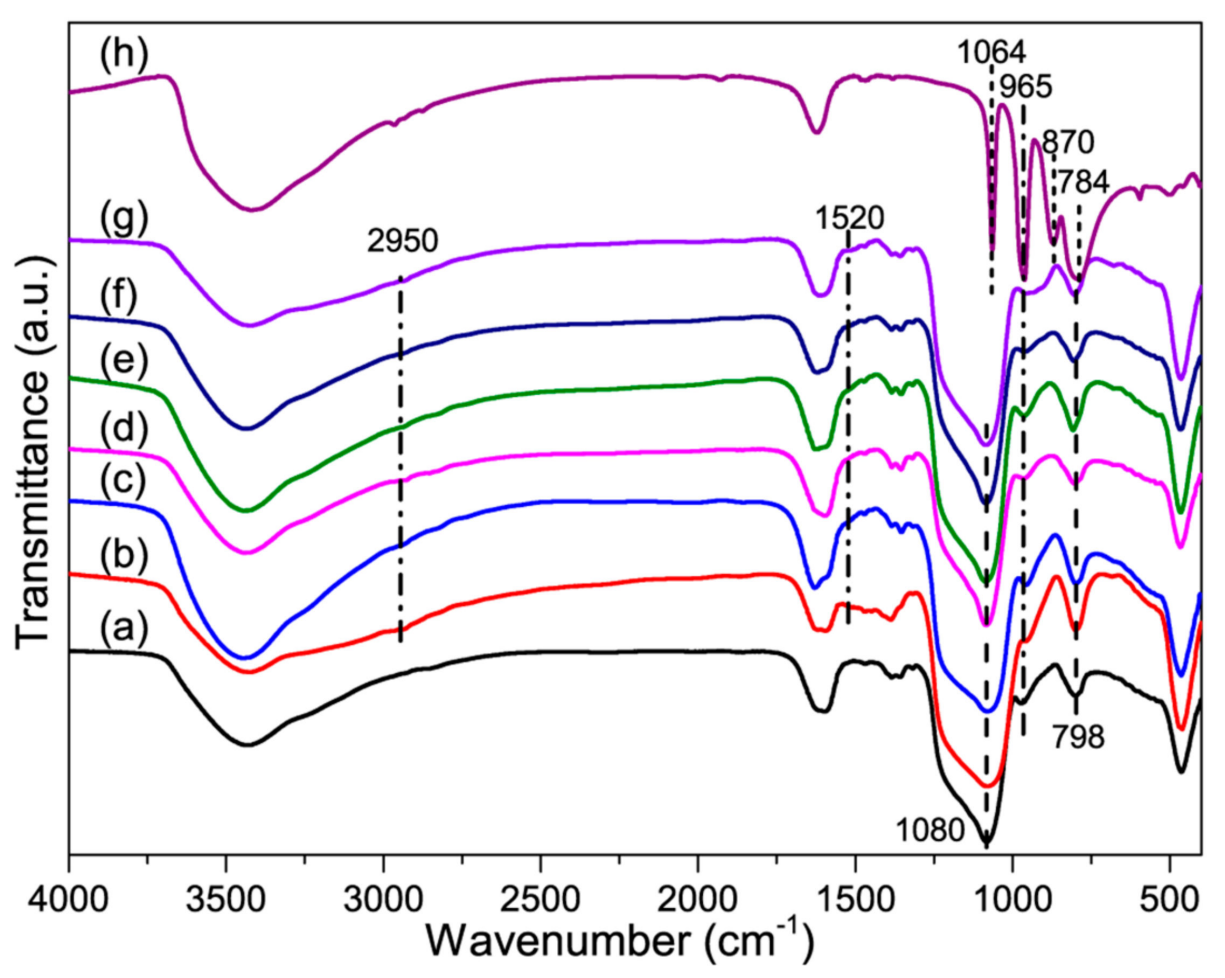
Fourier transform infrared (FT-IR) spectra of (**a**) SBA-15, (**b**) NH_2_-SBA-15, (**c**) 3.3PMo/NH_2_-SBA-15, (**d**) 6.7PMo/NH_2_-SBA-15, (**e**) 10PMo/NH_2_-SBA-15, (**f**) 13.3PMo/NH_2_-SBA-15, (**g**) 16.7PMo/NH_2_-SBA-15, and (**h**) H_3_PMo_12_O_40_·nH_2_O.

Figure 1 shows the FT-IR spectra of SBA-15, NH_2_-SBA-15 and xPMo/NH_2_-SBA-15 catalysts. A broad band ranging from 3000 to 3600 cm^−1^ and a band around 1620 cm^−1^ observed for all samples is attributed to the vibration of O–H of physisorbed water [34,35]. Compared with SBA-15, NH_2_-SBA-15 had two new peaks at 2950 and 1520 cm^−1^, which were assigned to the stretching mode of C–H and bending mode of N–H, respectively, indicating that the aminopropyl groups were successfully grafted on the surface of SBA-15 [34,36]. The decreased intensity of Si–OH band around 965 cm^−1^ also implies the successful amine functionalization [37]. As for SBA-15 or NH_2_-SBA-15, the bands at 1080 and 798 cm^−1^ were attributed to the symmetric and asymmetric stretching vibration of Si–O–Si, respectively [38]. The typical Keggin structure of PMo was identified by four characteristic bands in the range of 1300–600 cm^−1^. The band at 1064 cm^−1^ was attributed to the asymmetric stretching vibration of P–O_t_, and the bands at 965, 870, and 784 cm^−1^ were attributed to the stretching modes of terminal Mo=O_t_, edge sharing of Mo–O_b_–Mo, and corner sharing of Mo–O_c_–Mo, respectively [39]. For xPMo/NH_2_-SBA-15 catalysts, the four characteristic bands of PMo were overlapped by the strong bands of Si–O–Si and Si–OH of NH_2_-SBA-15. However, the peak width of the band ranging from 1000 to 860 cm^−1^ increased with the increase of amount of PMo, indicating the successful immobilization of PMo on the surface of NH_2_-SBA-15.

#### 3.1.2. XRD

**Figure 2:**
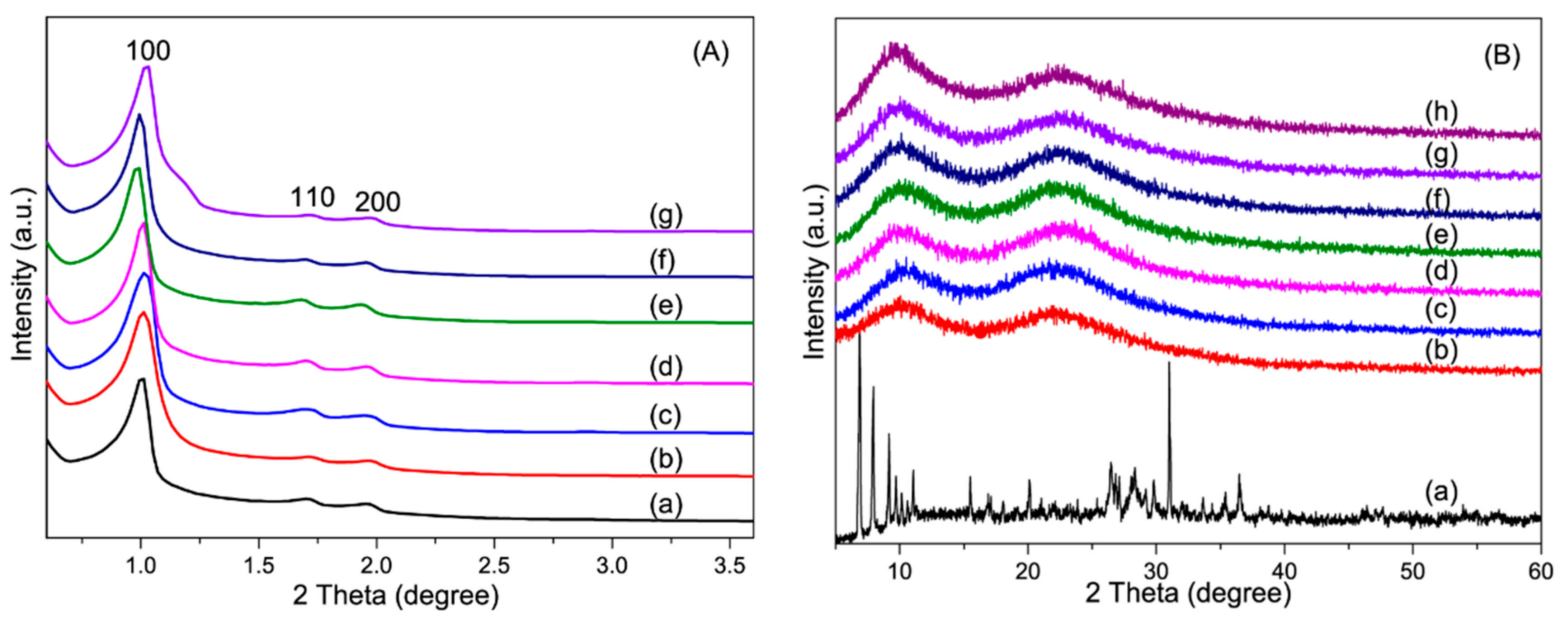
(**A**) Small-angle XRD patterns of (a) SBA-15, (b) NH_2_-SBA-15, (c) 3.3PMo/NH_2_-SBA-15, (d) 6.7PMo/NH_2_-SBA-15, (e) 10PMo/NH_2_-SBA-15, (f) 13.3PMo/NH_2_-SBA-15, and (g) 16.7PMo/NH_2_-SBA-15; (**B**) Wide-angle XRD patterns of (a) H_3_PMo_12_O_40_·nH_2_O, (b) SBA-15, (c) NH_2_-SBA-15, (d) 3.3PMo/NH_2_-SBA-15, (e) 6.7PMo/NH_2_-SBA-15, (f) 10PMo/NH_2_-SBA-15, (g) 13.3PMo/NH_2_-SBA-15, and (h) 16.7PMo/NH_2_-SBA-15.

The small-angle XRD patterns of SBA-15, NH_2_-SBA-15 and xPMo/NH_2_-SBA-15 catalysts are depicted in Figure 2A. Three diffraction peaks, one strong peak around 1° attributed to (100) plane and two weak peaks around 1.7° and 1.9° attributed to (110) and (200) planes, respectively, are shown for all the samples [32]. It suggests that the primary two-dimensional hexagonal structure of SBA-15 was maintained after the modification with APTES and the further immobilization of PMo. Compared with SBA-15 and NH_2_-SBA-15, no diffraction peak of PMo was detected in the wide-angle XRD patterns of xPMo/NH_2_-SBA-15, indicating the high dispersion of PMo species on the support [36].

#### 3.1.3. Nitrogen Adsorption–Desorption

N_2_ adsorption–desorption isotherms of SBA-15, NH_2_-SBA-15 and xPMo/NH_2_-SBA-15 samples are shown in Figure S1 and the textural properties are listed in Table 1. All the samples had typical type IV isotherms and H1 type hysteresis loops, indicating the uniform mesoporous structures [40]. After the modification with APTES, the BET surface area decreased from 599 to 343 m^2^·g^−1^, and the pore volume declined from 0.80 to 0.54 cm^3^·g^−1^. The pore size calculated from the isotherm also reduced from 5.5 to 5.1 nm. When PMo was immobilized on NH_2_-SBA-15, the surface area showed a gradually decreased trend with increased amount of PMo. A similar phenomenon was observed for pore volume. However, the introduction of PMo had little effect on the pore size due to low loading of phosphomolybdic acid.

#### 3.1.4. ^31^P MAS NMR Spectroscopy

**Figure 3:**
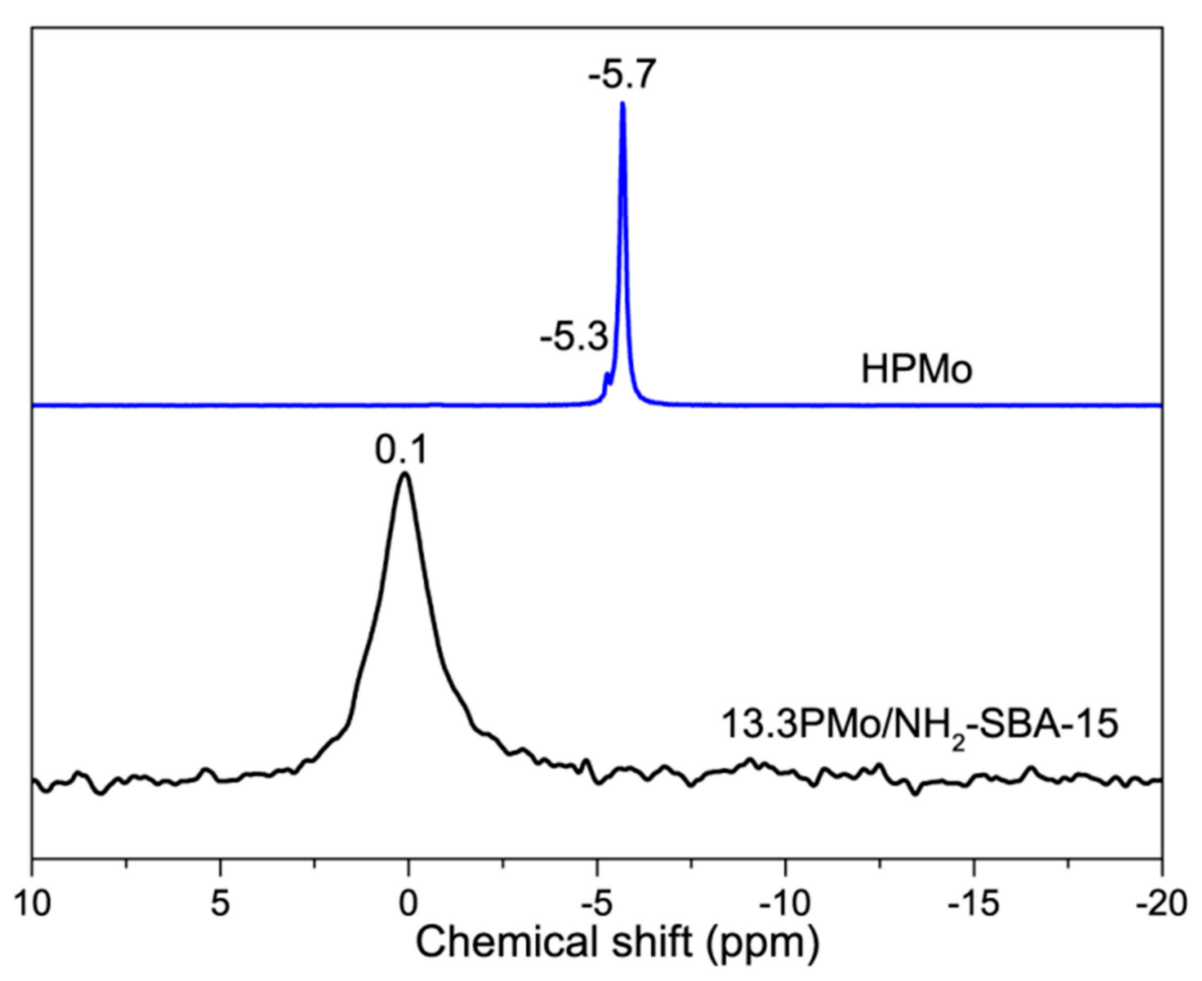
^31^P MAS NMR spectra of H_3_PMo_12_O_40_·nH_2_O and 13.3PMo/NH_2_-SBA-15.

As displayed in Figure 3, ^31^P MAS NMR spectrum of HPMo showed a strong resonance at −5.7 ppm and a weak resonance at −5.3 ppm. In the case of 13.3PMo/NH_2_-SBA-15 catalyst, there was a single resonance at 0.1 ppm, further confirming the successful immobilization of PMo species. The broadening of NMR peak and shifting to down-field of 0.1 ppm were related to the decrease of physisorbed and structural water during immobilization [41]. Also, the strong interaction between PMo and NH_2_-SBA-15 may also have been responsible for the shift of NMR peak [42].

### 3.2. Catalytic Epimerization of Aldoses

#### 3.2.1. Catalytic Activity of xPMo/NH_2_-SBA-15 for Glucose Epimerization

The catalytic activity of various catalysts was investigated in the glucose epimerization. As shown in Table 2, no glucose conversion could be found in the blank experiment without catalyst or only with the bare SBA-15. When NH_2_-SBA-15 was used as a catalyst, the conversion of glucose was 8.9%, and the selectivity of fructose and mannose were 38.2% and 5.6%, respectively. According to the research of Carraher et al. [43], OH^-^ generated from the hydrolysis of –NH_2_ group can simultaneously catalyze the isomerization and epimerization of glucose, with the isomeric fructose being the main product [43]. As a result, a little fructose was observed when NH_2_-SBA-15 was used as the catalyst. In addition, a large number of side reactions existed in the base catalyst system, and these products could not be precisely analyzed by HPLC. The color of the solid catalysts changed from white to dark brown after the reaction, indicating that Maillard reaction occurred in the system [44]. When the PMo polyanions were introduced to NH_2_-SBA-15 via the neutralization of –NH_2_ groups with H^+^ of HPMo during the preparation process [45], no fructose was observed for the xPMo/NH_2_-SBA-15 catalysts. The glucose conversion increased from 6.8% over 3.3PMo/NH_2_-SBA-15 to 34.8% over 13.3PMo/NH_2_-SBA-15 and the selectivity of mannose increased dramatically from 29.4% to 85.6%. The mannose yield of 29.8% is close to the theoretical equilibrium yield reported in the literature [28,46]. The glucose conversion continuously increased with the decrease of mannose selectivity over 16.7PMo/NH_2_-SBA-15, attributing to the enhanced side reactions. These results reveal that the PMo is of great significance for the epimerization of glucose. The catalytic performance of 13.3PMo/NH_2_-SBA-15 was superior to that of Sn-β zeolite or porous tin-organic frameworks and comparable to that of layered niobium molybdates in the aqueous reaction system [16,17,18,28,47].

#### 3.2.2. Calculation of the Activation Energy

**Figure 4:**
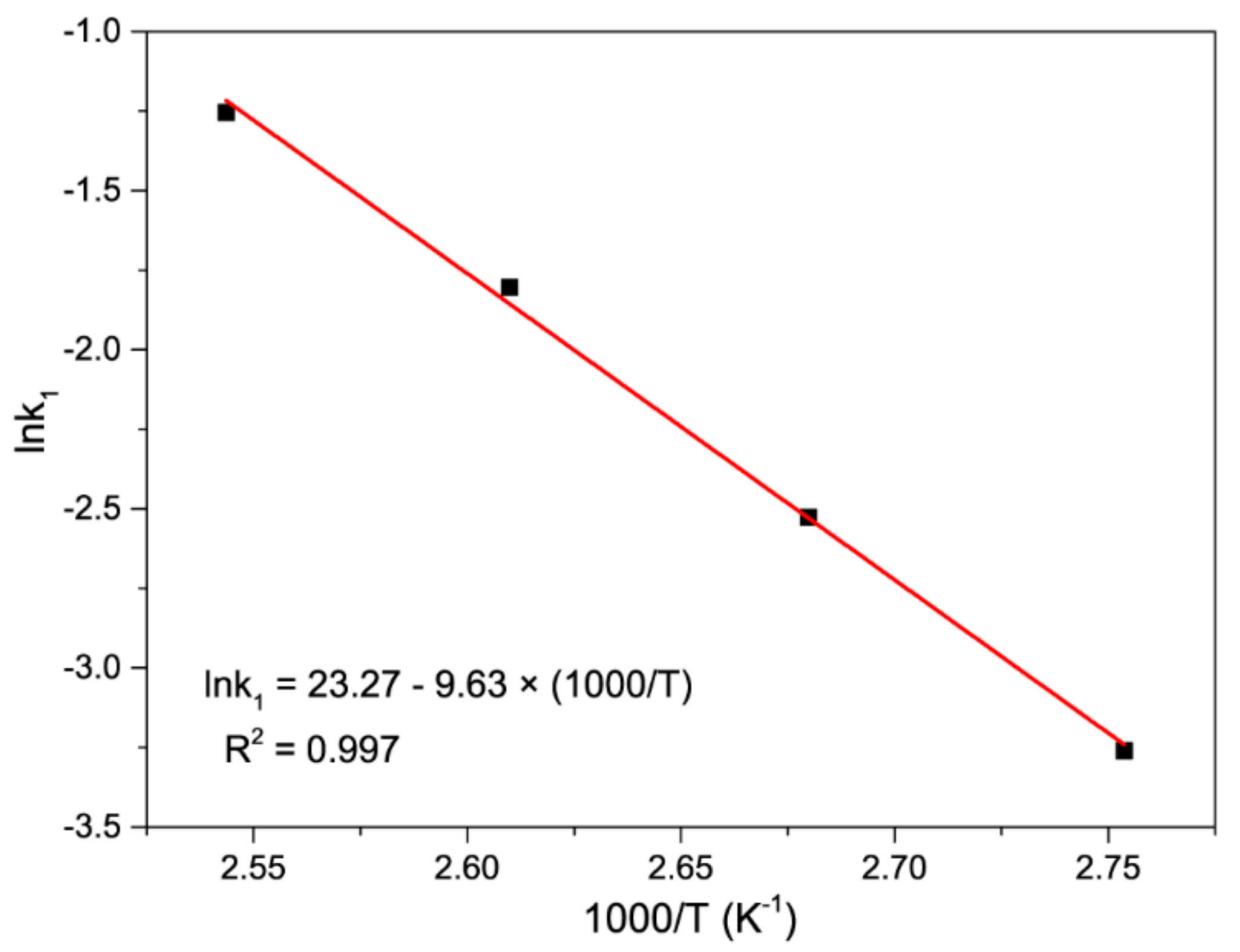
Arrhenius plot for epimerization of glucose over 13.3PMo/NH_2_-SBA-15.

Experiments with controlled reaction temperature were conducted to determine the activation energy (Table S1 in the Supplementary Information). Based on the assumption that the epimerization of glucose is a first order reaction, the apparent activation energy (E_a_) of the reaction over 13.3PMo/NH_2_-SBA-15 was calculated to be 80.1 ± 0.1 kJ·mol^−1^ (Figure 4), which is lower than the 96 kJ·mol^−1^ over homogeneous PMo catalyst [29]. Rellan-Pineiro et al. [48] reported that the reducibility of Mo atoms on the surface Mo-based catalysts plays an important role in the process of 1,2 carbon shift. The strong interaction between PMo species and the support in 13.3PMo/NH_2_-SBA-15 may influence the reducibility of the Mo atom, so that the glucose molecule is more easily be transformed into a transition state.

#### 3.2.3. Catalytic Activity of 13.3PMo/NH_2_-SBA-15 for the Reverse Reaction

**Figure 5:**
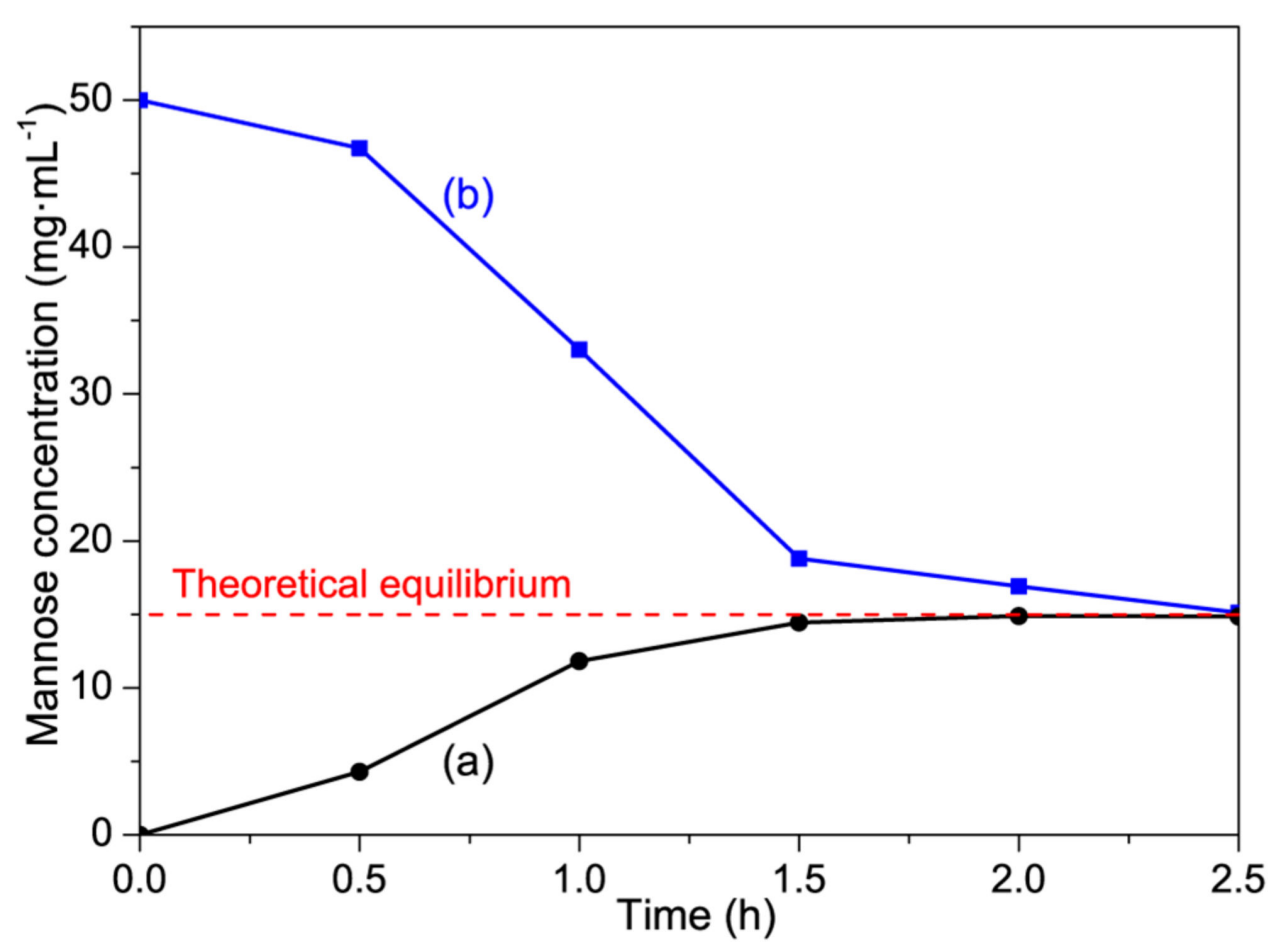
Time courses of epimerization of (**a**) glucose and (**b**) mannose over 13.3PMo/NH_2_-SBA-15. Reaction conditions: 40 mg of catalyst, 5 mL of 5 wt% aldose aqueous solution, 120 °C.

13.3PMo/NH_2_-SBA-15 catalyst may not only catalyze the epimerization reaction of glucose, but also catalyze its reverse reaction. Figure 5 shows the time courses of mannose concentration in epimerization reaction of glucose and its reverse reaction of mannose epimerization. It is clearly shown that mannose concentration in the glucose epimerization reached theoretical equilibrium at a shorter time than that in the reverse reaction. This phenomenon is consistent with the research of Ju et al. [29] over HPMo catalyst. Hayes et al. [49] reported that some unreactive complexes could be formed in the system of mannose, which did not epimerize to glucose.

#### 3.2.4. Reusability of the Catalyst for Glucose Epimerization

**Figure 6:**
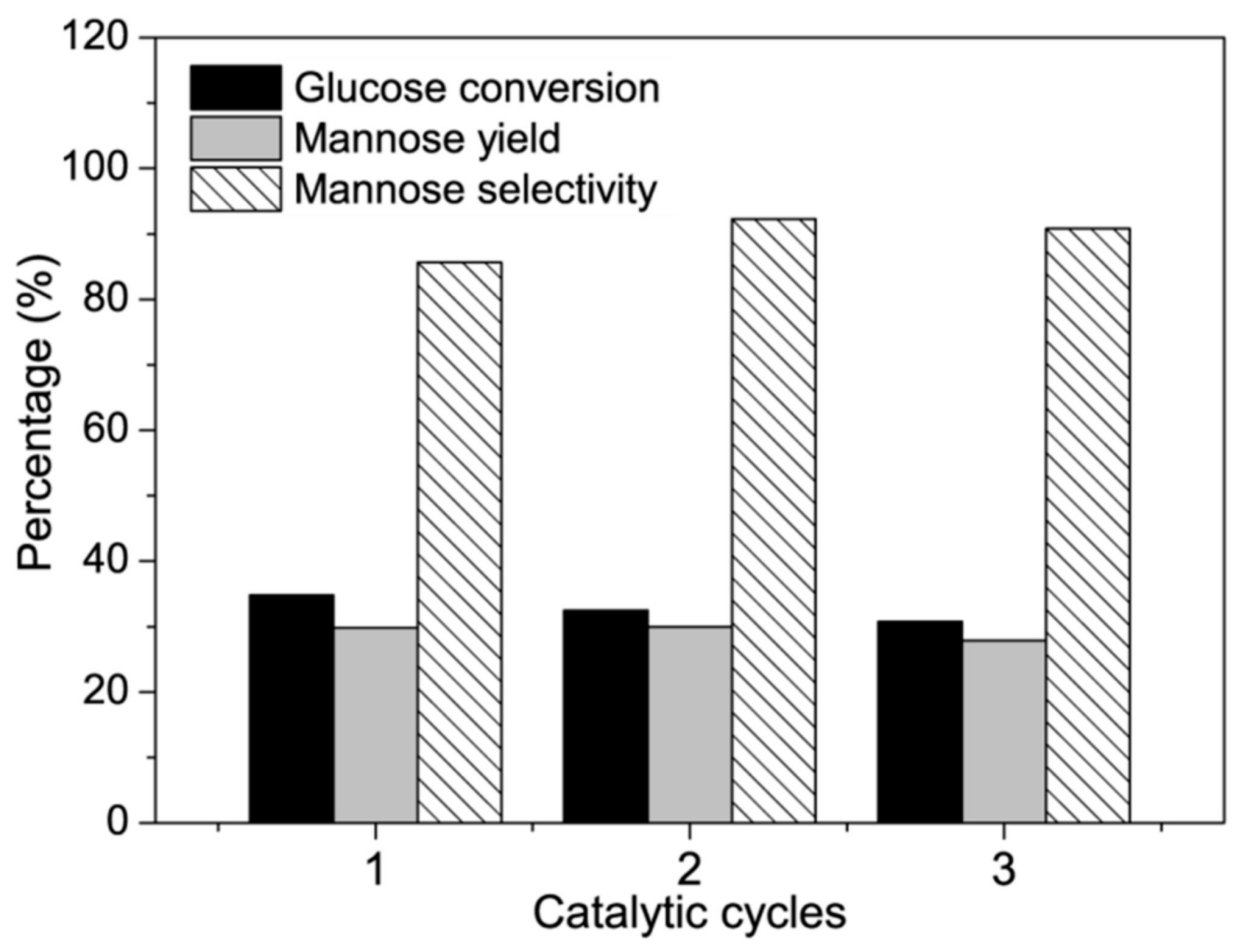
Recycling experiments over 13.3PMo/NH_2_-SBA-15. Reaction conditions: 40 mg of recycled catalyst, 5 mL of 5 wt% glucose solution, 120 °C, 2 h.

In order to investigate the reusability of the catalyst, 13.3PMo/NH_2_-SBA-15 catalyst with the best catalytic activity was chosen for recovery experiments. After each reaction, the catalyst was separated by centrifugation, washed three times with water and dried at 60 °C, and then used again for the glucose epimerization. As shown in Figure 6, the 13.3PMo/NH_2_-SBA-15 exhibited no significant decrease of the catalytic activity after three cycles in glucose epimerization. As anticipated, no apparent leaching of PMo species (<0.1%) occurred during the reaction as confirmed by ICP-AES tests, which is much less than that in the reported system catalyzed by Ag_3_PMo_12_O_40_. These results may be related to the strong interaction between PMo and NH_2_-SBA-15. Compared with insoluble salts of PMo, immobilization of the PMo on amine functionalized support may have been more beneficial for heterogenization of HPMo in the epimerization of glucose.

#### 3.2.5. Catalytic Activity of 13.3PMo/NH_2_-SBA-15 for Other Aldoses Epimerization

**Scheme 2:**
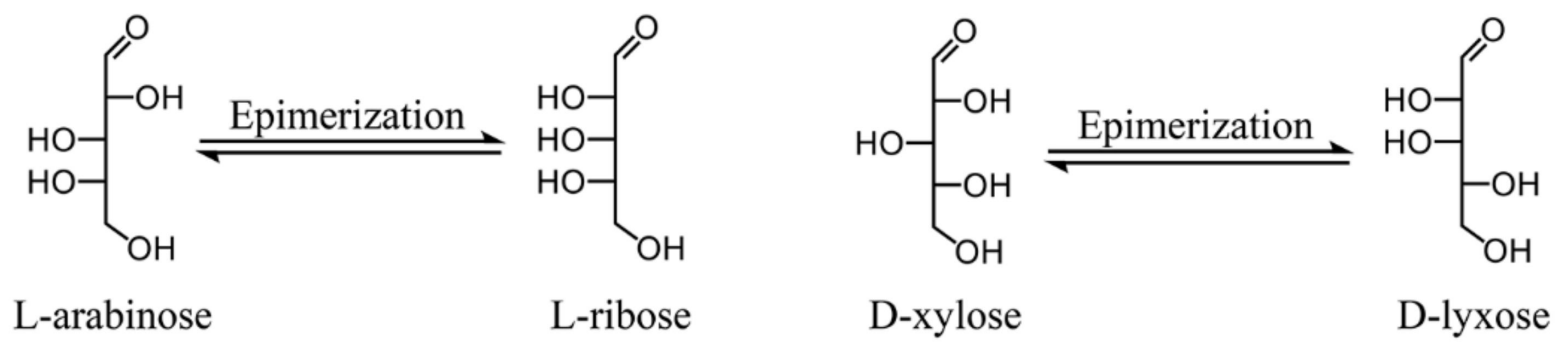
The epimerization of arabinose and xylose.

We also investigated the catalytic activity of the 13.3PMo/NH_2_-SBA-15 catalyst for the epimerization of other aldoses at the C-2 position (Scheme 2). As shown in Table 3, the arabinose conversion and ribose yield were 27.7% and 15.9%, respectively. The xylose conversion and the lyxose yield were 44.6% and 33.3%, respectively. Similar to glucose epimerization, these two reactions were also limited by thermodynamics, with arabinose/ribose and xylose/lyxose theoretical equilibrium ratios being 69:31, 67:33, respectively, according to the Gibbs free energy calculations [28]. The yield of lyxose was close to the equilibrium yield, but the selectivities of the epimeric aldoses in the both reaction systems were lower than mannose due to the presence of more side reactions. As a result, besides C6 aldoses, the C5 aldoses, like arabinose and xylose could also be effectively transformed into their C-2 epimers over the 13.3PMo/NH_2_-SBA-15 catalyst.

## 4. Conclusions

A series of xPMo/NH_2_-SBA-15 heterogeneous catalysts were prepared and tested in the glucose epimerization. The reaction over 13.3PMo/NH_2_-SBA-15 reached almost equilibrium within 2 h with a glucose conversion of 34.8% and mannose yield of 29.8%. The activation energy of the reaction over 13.3PMo/NH_2_-SBA-15 was lower than that over the homogeneous HPMo catalyst. The leaching of PMo was negligible in the reaction system. Mannose, arabinose and xylose could also be transformed to their corresponding C-2 epimeric aldoses successfully over the catalyst. Our work provides a promising heterogeneous catalyst for the epimerization of aldoses in aqueous solution.

## Figures and Tables

**Table 1:** Textural properties of SBA-15, NH_2_-SBA-15 and xPMo/NH_2_-SBA-15.

Samples	Nitrogen Content ^a^ (wt%)	Molybdenum Content ^b^ (wt%)	S_BET_ (m^2^·g^−1^)	D_pore_ (nm)	V_pore_ (cm^3^·g^−1^)
SBA-15	-	-	599	5.5	0.80
NH_2_-SBA-15	1.6	-	343	5.1	0.54
3.3PMo/NH_2_-SBA-15	1.1	0.6	337	5.1	0.53
6.7PMo/NH_2_-SBA-15	1.1	1.4	326	5.1	0.53
10PMo/NH_2_-SBA-15	1.1	3.0	304	5.1	0.51
13.3PMo/NH_2_-SBA-15	1.2	5.4	286	5.1	0.50
16.7PMo/NH_2_-SBA-15	1.1	6.3	267	5.1	0.46

^a^ Analyzed by elemental analyzer; ^b^ Analyzed by ICP-AES.

**Table 2:** Catalytic performance of the catalysts for glucose epimerization. ^a^

Entry	Catalyst	Glucose Conversion (%)	Mannose Yield ^b^ (%)	Mannose Selectivity (%)	Fructose Yield (%)	Fructose Selectivity (%)
1	−^c^	0	0	0	0	0
2	SBA-15	0	0	0	0	0
3	NH_2_-SBA-15	8.9 ± 0.1	0.5 ± 0.1	5.6	3.4 ± 0.1	38.2
4	3.3PMo/NH_2_-SBA-15	6.8 ± 0.2	2.0 ± 0.1	29.4	0	0
5	6.7PMo/NH_2_-SBA-15	7.4 ± 0.1	3.8 ± 0.1	51.4	0	0
6	10PMo/NH_2_-SBA-15	27.2 ± 0.2	22.9 ± 0.3	84.2	0	0
7	13.3PMo/NH_2_-SBA-15	34.8 ± 0.2	29.8 ± 0.1	85.6	0	0
8	16.7PMo/NH_2_-SBA-15	35.3 ± 0.1	29.7 ± 0.1	84.1	0	0

^a^ Reaction conditions: 40 mg of catalyst, 5 mL of 5 wt% glucose solution, 120 °C, 2 h; ^b^ Equilibrium yield of mannose is theoretically calculated to be 30%; ^c^ The blank experiment without catalyst.

**Table 3:** Catalytic performance of the catalysts for aldose epimerization. ^a^

Entry	Sugar	Conversion (%)	Epimer Yield (%)	Epimer Selectivity (%)
1	Glucose	34.8 ± 0.2	29.8 ± 0.1	85.6
2	Mannose	66.2 ± 0.1	60.5 ± 0.1	91.4
3	Arabinose	27.7 ± 0.2	15.9 ± 0.3	57.4
4	Xylose	44.6 ± 0.1	33.3 ± 0.2	74.7

^a^ Reaction conditions: 40 mg of catalyst, 5 mL of 5 wt% aldose solution, 120 °C, 2 h.

## Supplementary Materials

The following are available online at https://www.mdpi.com/1996-1944/13/3/507/s1, Figure S1. N_2_ adsorption-desorption isotherms of (a) SBA-15, (b) NH_2_-SBA-15, (c) 3.3PMo/NH_2_-SBA-15, (d) 6.7PMo/NH_2_-SBA-15, (e) 10PMo/NH_2_-SBA-15, (f) 13.3PMo/NH_2_-SBA-15, and (g) 16.7PMo/NH_2_-SBA-15, Table S1: Catalytic performance of 13.3PMo/NH_2_-SBA-15 for glucose epimerization.
