# Supplementary material for: H3PMo12O40 Immobilized on Amine Functionalized SBA-15 as a Catalyst for Aldose Epimerization
Source: Materials (Basel). 2020 Jan 21;13(3):507. doi: 10.3390/ma13030507 (PMC7040683; doi:10.3390/ma13030507)
Supplement: Supplementary file 1 [file materials-13-00507-s001.pdf]

Supplementary Information

$H_3PMo_{12}O_{40}$ immobilized on amine functionalized SBA-15 as a catalyst for aldose epimerization

Hui Wang, Meiyin Wang, Jining Shang, Yuanhang Ren, Bin Yue* and Heyong He*

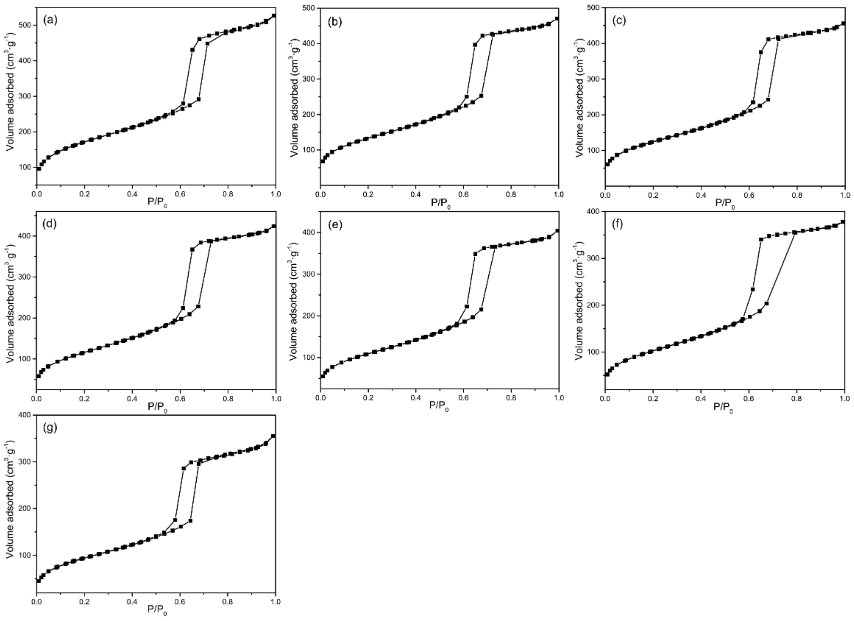

Figure S1. N_2 adsorption-desorption isotherms of (a) SBA-15, (b) NH_2-SBA-15, (c) 3.3PMo/NH_2-SBA-15, (d) 6.7PMo/NH_2-SBA-15, (e) 10PMo/NH_2-SBA-15, (f) 13.3PMo/NH_2-SBA-15, and (g) 16.7PMo/NH_2-SBA-15.

Table S1. Catalytic performance of 13.3PMo/NH_2-SBA-15 for glucose epimerization.[a]

Entry	Temperature (°C)	Glucose conversion (%)	Mannose yield (%)	Mannose selectivity (%)
1	90	5.6±0.1	3.5±0.2	62.3
2	100	11.3±0.1	8.1±0.2	71.7
3	110	21.9±0.1	18.3±0.1	83.6
8	120	34.8±0.2	29.8±0.1	85.6

[a]Reaction conditions: 40 mg of catalyst, 5 mL of 5 wt% glucose solution, 2 h.
